# Early Detection of Poor Outcome after Mild Traumatic Brain Injury: Predictive Factors Using a Multidimensional Approach a Pilot Study

**DOI:** 10.3389/fneur.2017.00666

**Published:** 2017-12-12

**Authors:** Sophie Caplain, Sophie Blancho, Sébastien Marque, Michèle Montreuil, Nozar Aghakhani

**Affiliations:** ^1^Laboratory of Psychopathology and Neuropsychology, University Paris 8, Saint-Denis, France; ^2^Institut pour la Recherche sur la Moelle Epinière et l’Encéphale, Paris, France; ^3^Capionis Research, Bordeaux, France; ^4^Department of Neurosurgery, Bicêtre University Hospital, Assistance Publique Hôpitaux de Paris, Le Kremlin-Bicêtre, France

**Keywords:** mild traumatic brain injury, assessment score, human, post-concussion syndrome, prognostic factors

## Abstract

Mild traumatic brain injury (MTBI) is a common condition within the general population, usually with good clinical outcome. However, in 10–25% of cases, a post-concussive syndrome (PCS) occurs. Identifying early prognostic factors for the development of PCS can ensure widespread clinical and economic benefits. The aim of this study was to demonstrate the potential value of a comprehensive neuropsychological evaluation to identify early prognostic factors following MTBI. We performed a multi-center open, prospective, longitudinal study that included 72 MTBI patients and 42 healthy volunteers matched for age, gender, and socioeconomic status. MTBI patients were evaluated 8–21 days after injury, and 6 months thereafter, with a full neurological and psychological examination and brain MRI. At 6 months follow-up, MTBI patients were categorized into two subgroups according to the Diagnostic and Statistical Manual of Mental Disorders (DSM-IV) as having either favorable or unfavorable evolution (UE), corresponding to the presence of major or mild neurocognitive disorder due to traumatic brain injury. Univariate and multivariate logistical regression analysis demonstrated the importance of patient complaints, quality of life, and cognition in the outcome of MTBI patients, but only 6/23 UE patients were detected early via the multivariate logistic regression model. Using several variables from each of these three categories of variables, we built a model that assigns a score to each patient presuming the possibility of UE. Statistical analyses showed this last model to be reliable and sensitive, allowing early identification of patients at risk of developing PCS with 95.7% sensitivity and 77.6% specificity.

## Introduction

Approximately 80,000 new cases of mild traumatic brain injury are admitted to emergency units in France annually. Although most patients recover completely within weeks or months, approximately 10–25% (1–13) will have persisting symptoms with social and vocational consequences that appear disproportionate to the severity of the initial neurologic trauma (10). Such findings are now known as post-concussive syndrome (PCS). Symptoms include somatic, cognitive, and emotional complaints such as headache, sleep disturbances, balance disorders, cognitive impairment, fatigue, and mood or behavioral disorders.

Such persisting symptoms can affect patient outcomes in all aspects of life, with significant consequences with regard for public health (14–16).

Over the past decade, investigators have highlighted the role of predictive factors for the development and persistence of PCS. These factors can be pre-traumatic, related to the trauma itself, or post-traumatic. According to a critical review of the literature by Carroll and colleagues (WHO, Collaborating Centre Task Force on Mild Traumatic Brain Injury, 2004) (8), factors predicting PCS (with variable degrees of evidence) are divided into three categories:

1. The person: female gender, married status, enrolled in school, age over 40 years, pre-existing physical handicap, prior cerebral disease or neurological problem, prior head injuries, psychiatric problems, and major life stressors.
2. Injury: motor vehicle collision, responsibility.
3. Consequences: Glasgow Coma Scale (GCS) <15, loss of consciousness, post-traumatic amnesia (PTA) >20 min, experiencing post-injury nausea or memory problems, other injuries.

Seeking compensation and/or litigation were identified as important factors in patients with persisting symptoms.

Most recently, Cassidy and co-workers showed that more acute symptoms, poorer premorbid mental and physical health, and a major acute life stressor could predict persistent symptoms (13). Ponsford and associates also demonstrated the importance of premorbid psychiatric problems such as anxiety (17). However, literature pertaining to prognosis following MTBI varies substantially in quality, with multiple biases identified (8). The identification of corroborated risk factors could allow earlier and better-tailored treatment plans, and potentially decrease the incidence of persistent PCS.

We wished to demonstrate the potential value of a comprehensive neuropsychological complaints and quality of life (QoL) evaluation to identify early prognostic factors for MTBI patients, and to establish short clinical assessment tools applicable during the early stage of MTBI for patients susceptible to develop PCS.

## Materials and Methods

### Patients

Two groups were assessed: patients diagnosed with MBTI, and healthy volunteers. Patients had been diagnosed with MBTI according to established criteria of the American Congress Of Rehabilitation Medicine (Mild Traumatic Brain Injury Committee) in 1993 (18), which requires the presence of at least one of the following symptoms: initial loss of consciousness <30 min; PTA lasting <24 h; a GCS score of 13–14 at time of injury; a GCS of 13–15 after 30 min; altered mental state at the time of the accident (e.g., confusion, disorientation, etc.); or a focal neurological deficit that may or may not be transient.

Patients were ineligible if they were intubated, ventilated, or sedated on arrival at hospital; presented a spinal cord injury with neurological symptoms or disabling multiple injuries with at least one injury considered life-threatening; presented a head injury after a suicide attempt; had psychiatric or psychological disorders that were either disabling or might interfere with follow-up; featured psychotropic medication intake at the time of injury; had a history of hospitalization in a specialized psychiatric unit and/or sick leave for psychological reasons; had a history of severe head injury; had a progressive neurological disease; presented with a drug or alcohol addiction, were under guardianship; or had a contraindication to MR imaging.

The control group was composed of healthy people without any history of head injury.

Individuals in both groups had to be between 18 and 60 years of age.

### Study Design

A multi-center, open, prospective, longitudinal study was performed. Eighty-six consecutive MTBI patients were enrolled from the emergency departments of two Parisian academic hospitals (Bicêtre and Bichat). The control group was composed of 42 healthy volunteers, matched for age, gender, and socioeconomic status with the MBTI group.

**Figure 1:**
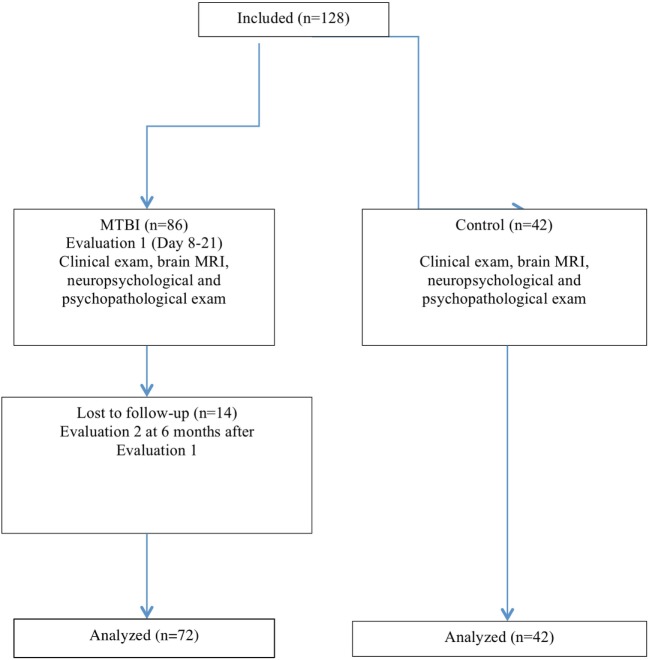
Flow diagram of subject recruitment and evaluation.

Mild traumatic brain injury patients were assessed between 8 and 21 days after the injury, and again 6 months later. Patients underwent a full clinical neurological examination, a brain MRI, neuropsychological and psychological evaluations at two separate points in time. Neurological examinations and psychological assessments were performed in the neurosurgical unit of the Bicêtre University hospital. At the 6-month follow-up visit, we used the DSM-IV international criteria to categorize MBTI patients into two subgroups. Patients were classified as having favorable evolution (FE) or unfavorable evolution (UE), an unfavorable classification corresponding to the presence of PCS due to traumatic brain injury (Figure 1). The study was approved by the Ethics Committee of the Pitié-Salpêtrière University Hospital (ID RCB: 2008-A01542-53, Paris, France). All patients provided informed consent prior to any study procedure.

### Evaluations

Data were collected for demographics, circumstances of the accident, and medical history. Standardized and classical neuropsychological tests, psychopathological scales, QoL questionnaires and Visual Analog Scale (VAS) evaluation, pain (headache and other), and complaints are described below and summarized in Table 1.

**Table 1:** Summary of domains evaluated, tests, scales/questionnaires used, and parameters collected.

Functions/dimensions evaluated	Tests and scales
**Neuropsychological evaluations**	
Short-term and working memory	– Verbal memory span (direct order) – Verbal memory span (reverse order) – Letter/number sequence MEM III (Wechsler Memory Scale)

Spontaneous flexibility	– Verbal semantic and phonemic fluency (“animals” and letter “M”) – 1-min version (21)

Reactive flexibility	– Trail Making Test A, Trail Making Test B

Inhibition	– Stroop test (20)

Speed of processing information/maintenance of attention	– Paced Auditory Serial Addition Test

Selective/divided attention	– Concentrated attention test d2 (22)

**Questionnaires and scales**	
Anxiety/depression	– Anxiety and depression scale, Hospital Anxiety Depression Scale – STAI-A (French version) (23)

Quality of life (QoL)	– Visual Analog Scale (VAS) global QoL – Quality of Life After Brain Injury Questionnaire (26)

Pain	– Pain intensity (VAS) (headache and other pain)

Complaints	– Rivermead post-concussion questionnaire (RPCQ)

#### Neuropsychological Tests and Scoring

Short-term memory was evaluated using verbal memory span according to the Wechsler Memory Scale (MEM III) as well as working memory according to reverse verbal memory span. Executive functions were tested using the Trail Making Test A (TMT A) and B (TMT B) (19). Inhibition was evaluated with the Stroop test, 45-s version (20). Information treatment speed was evaluated with the Paced Auditory Serial Addition Test (PASAT), which also solicits executive functions (resistance to interference, maintenance, and manipulation of working memory), and maintenance and sharing of attentional resources. Verbal semantic and phonemic fluency (“animals” and letter “M,” 1-min version) was used (21) and selective motor–visual attention capacities were evaluated using the d2 Test (22).

Two cognitive composite scores were calculated at each time point. A low-level treatment score was calculated that took into account attention maintenance scores, verbal memory span (direct order), TMT A, Stroop words reading (W), and Stroop color naming (C) tests. A high-level treatment score was calculated that took into account complex information’s treatments, including verbal working memory span (reverse order), TMT B-A score, Stroop words/color naming (W/C) test, PASAT correct answer (CA) and telescoping error (TE), verbal phonemic fluency “M.”

#### Psychopathological Scales

Mood evaluation scales were used to evaluate the presence of anxiety or depression syndromes. We employed the State-Trait Anxiety Inventory A self-evaluation scale (STAI-A Forms Y-A and Y-B, French version) (23), along with the Hospital Anxiety Depression Scale (24) to assess for anxiety. The presence of a serious depressive syndrome according to DSM-IV criteria was also evaluated following a semi-structured interview (25).

#### Quality of Life

Global QoL was evaluated using a visual analogic scale (VAS, maximum value of 10) on which subjects had to indicate their degree of satisfaction in life overall (all areas combined) for the 15 days prior to the evaluation. An in-depth evaluation was performed using the Quality of Life After Brain Injury (QOLIBRI) QoL questionnaire (26), which is specifically adapted to brain injury patients and is designed to evaluate QoL in all domains of daily life (cognitive, physical, social, emotional, and personal) and validated in French.

#### Pain

The intensity of headaches and other pain was evaluated using VAS.

#### Complaints

We employed the Rivermead Post-Concussion Symptoms Questionnaire (RPCQ) (27). It is adapted to MTBI patients and evaluates 16 major complaints reported in PCS, including the intensity of the complaint.

#### MRI

Neuroimaging data were reported previously (28, 29).

Mild traumatic brain injury patients with post-concussion syndrome had greater alterations than patients without post-concussion syndrome. In patients with post-concussion syndrome, changes specifically affected temporal and thalamic regions predominantly at the subacute stage and frontal regions at the late phase. Our results suggest that the post-concussion syndrome is associated with specific abnormalities in functional brain network that may contribute to explain deficits typically observed in PCS patients.

### Statistical Analysis

Statistical analysis was performed using SAS^®^ V9.3 software. Descriptive and inferential analyses were performed. Demographic, medical, test, and scale data were compared between the two MTBI subgroups (FE and UE), as well as between the two subgroups and the control group at the two-time points.

For each group, outcomes between the two time points were assessed using parametric tests (Student’s *t*-test and the chi-squared test) or non-parametric tests (Fisher’s exact for *N* < 5, and the Wilcoxon signed-rank test for *N* < 30). A univariate logistic regression model was performed on each variable to identify potential predictive factors in the favorable vs. unfavorable groups. Variables with *p* < 0.01 were included in a multivariate logistic regression model.

Variables were removed from the full model using a descending stepwise selection strategy and removing variables with *p* > 0.05 until all variables would be significant. The most appropriate model was defined as all variables significantly associated with the group. MTBI patients were classified as having UE or FE according to DSM-IV criteria.

For both univariate and multivariate, a goodness-of-fit assessment was performed in order to evaluate the robustness of modeling.

## Results

### Demographic Characteristics

A group of 86 MBTI patients was examined at the acute and subacute phase after the injury. 72 patients were evaluated after 6 months (fourteen patients were lost to follow-up). There were 50 men and 22 women, with a mean of 13 ± 5 days between the injury and the first evaluation, and 213 ± 53 days between the first and the second evaluation. The outcome was categorized as FE (i.e., without PCS) for 49 patients, and UE (i.e., with PCS) for 23 patients. Demographic, clinical, and injury characteristics are reported in Table 2.

**Table 2:** Demographic characteristics, type of accident, and initial clinical data.

		All	Favorable evolution	Unfavorable evolution	*p*-Value

		(*N* = 72)	(*N* = 49)	(*N* = 23)
Age, years	Mean (SD)	34.8 (11.3)	32.6 (11.0)	39.4 (10.7)	
Gender, *n* (%)	Male	50 (69.4)	37 (75.5)	13 (56.5)	
	Female	22 (30.6)	12 (24.5)	10 (43.5)	
Level of education^c^, *n* (%)	1 and 2	19 (26.4)	8 (16.3)	11 (47.8)	
	3	18 (25.0)	13 (26.5)	5 (21.7)	
	4	16 (22.2)	13 (26.5)	3 (13.0)	
	5	19 (26.4)	15 (30.6)	4 (17.4)	
**Type of accident**					
Attack		20 (27.8)	15 (30.6)	5 (21.7)^a^	0.114^a^
Fall		13 (18.1)	6 (12.2)	7 (30.4)	
Workplace accident		9 (12.5)	7 (14.3)	2 (8.7)	
Others		9 (12.5)	6 (12.2)	3 (13)	
Sporting accident		8 (11.1)	8 (16.3)	0 (0)	
Road accident					
Car		5 (6.9)	2 (4.1)	3 (13.0)	
Motorbike		4 (5.6)	2 (4.1)	2 (8.7)	
Bike		2 (2.8)	2 (4.1)	0 (0)	
Pedestrian		2 (2.8)	1 (2.0)	1 (4.3)	
Glasgow Coma Scale	14	2 (2.8)	1 (2.0)	1 (4.3)	0.540^a^
	15	70 (97.2)	48 (98.0)	22 (95.7)	
Initial loss of consciousness		27 (37.5)	16 (32.7)	11 (47.8)	0.218^b^
Post-traumatic amnesia		28 (38.9)	17 (34.7)	11 (47.8)	0.289^b^
Associated injury (s)		39 (54.2)	24 (49.0)	15 (65.2)	0.194^b^

*^a^Fisher’s exact test*.

*^b^Chi squared test*.

*^c^GREFEX criteria: 1 ≤ lower school certificate; lower school certificate < 2 < higher school certificate; 3, higher school certificate; 4, diploma; 5, higher education*.

*All data are presented as n (%)*.

No variable were reported as clinically or statistically significant between the two groups regarding medical history or neurological examination. Patients who were physically attacked were more likely to have a UE (*p* = 0.144). The UE group exhibited significantly poorer cognitive function than the EF group. Comparison of cognitive function between the two groups at the early time point demonstrated statistically significant differences for all variables evaluated, with the exception of the global MEM III verbal memory span (FE, mean 4.5 [SD 1.2]; UE, mean 3.9 [SD 1.2]; *p* = 0.059), working verbal memory based on letter/number sequences (FE, mean 8.5 [2.9]; UE, mean 7.0 [2.4]; *p* = 0.051), the TMT B-A score evaluating flexibility capacity (FE, mean 35.0 [18.5]; UE, mean 41.0 [20.5]; *p* = 0.221), and PASAT TE sub-score (FE, mean 1.3 [1.7]; UE, mean 2.8 [4.6]; *p* = 0.516). Levels of anxiety, depression, and pain (headache and other) were statistically higher in the UE group.

Unfavorable evolution exhibited lower global QoL (VAS, *p* < 0.001; QOLIBRI total score = 0.005). The QOLIBRI sub-scores did not exhibit any differences, although the level for each factor (physical condition, brain functioning, feelings and emotions, social and personal life) was lower for the unfavorable group. UE group also exhibited a significantly higher level of complaints than the favorable group for all items at the early time point (Table 3).

**Table 3:** Between-group comparison of complaints at the early evaluation (*p*-value).

	*p*-Value
Headache	0.004
Vertigo	0.001
Nausea and/or vomiting	<0.001
Noise intolerance	<0.001
Sleep disorders	0.011
Fatigue, need of sleep	<0.001
Irritability	<0.001
Depression, crying easily	<0.001
Sensation of frustration, impatience	<0.001
Memory loss	<0.001
Difficulty concentrating	<0.001
Slowed thinking	<0.001
Vision troubles	<0.001
Light sensitive	<0.001
Double vision	0.002
Agitation	<0.001
Total	<0.001^a^

*p Values are according to Fisher’s exact test, unless otherwise stated*.

*^a^Student’s t-test*.

### Development of a Prognostic Model

#### Univariate Logistic Regression Model

- All dimensions were linked to the prognosis of UE: cognitive, complaints, mood, somatic, and QoL domains.
- For those dimensions, mood and complaints were closely linked to prognosis and had a significant relationship to all evaluated variables. No specific group of cognitive variables was associated with UE.
- Only one QOLIBRI sub-score was not associated with prognosis in the QoL area (social and personal life, OR = 2.47 [95% CI, 0.89–6.85], *p* = 0.082).
- None of the demographic variables in the univariate logistic regression model demonstrated that gender and level of education [GREFEX criteria (30)] were not related to MTBI prognosis at 6 months (sex [OR] 0.42 [95% CI 0.15–1.21], *p* = 0.107; level of education 3, OR = 3.57 [95% CI 0.90–14.15], *p* = 0.070; level of education 4 OR = 5.96 [95% CI 1.26–28.10], *p* = 0.024; level of education 5, OR = 5.16 [95% CI 1.23–21.55], *p* = 0.025).
- In the medical data, only medicate treatment was connected with prognosis (OR = 0.09, [95% CI 0.03–0.29], *p* < 0.001).

#### Multivariate Logistic Regression Model

Considering the significant number of variables of interest, 20 most significant variables were selected (Table 4) (variables for which *p* < 0.001) among the 5 extracted domains in the univariate logistic regression model (cognitive, complaints, mood, somatic, and QoL) (Table 5) and were used in a multivariate logistic regression model. Three of these variables were kept from this model as significantly related to an unfavorable prognosis: complaint “concentrating difficulty”, VAS global QoL, and verbal phonemic fluency “M.” The size of the reliable interval likely reflects intrinsic patient heterogeneity and introduces uncertainty of these results in the clinical context (Table 6). This model identified as UE to T1 only 27% of the patients diagnosed UE to T2 by DSM-IV TR. There was no false positives, and none of the FE patients were identified as being at risk of UE at T2 (Table 7).

**Table 4:** List of 20 selected variables used in the multivariate logistic regression analysis.

Domain	Variables
Complaints (9)	Noise intolerance, easily bothered by noise
	Fatigue, great need for sleep
	Irritability, easily angered
	Depressed, cries easily
	Sensation of frustration, impatience
	Memory loss, memory difficulties
	Difficulty concentrating
	Slowed thinking
	Total number of symptoms

Mood (2)	HAD A
	HAD D

Pain (2)	Visual Analog Scale (VAS) headache
	VAS other pain

Quality of life (QoL) (4)	VAS global QoL
	Quality of Life after Brain Injury (QOLIBRI) physical condition
	QOLIBRI daily life
	QOLIBRI current and future situation

Neuropsychology (3)	Paced Auditory Serial Addition Test (PASAT) correct answer
	PASAT no response
	Verbal phonemic fluency “M”

**Table 5:** Multivariate logistic regression model (poor prognosis probability model).

		OR [95% CI]	*p*-Value

Model with neuropsychological scores (*N* = 60); 42 favorable evolution and 18 unfavorable evolution			
Complaints, difficulty concentrating	2–4 vs. 0–1	26.81 [3.75; 191.85]	0.0011
Visual Analog Scale global quality of life	≤5 vs. >5	9.56 [1.36; 67.06]	0.0230
Verbal phonemic fluency “M”	<10 vs. ≥10	12.33 [1.60; 95.03]	0.0159

**Table 6:** Multivariate logistic regression model results.

	Favorable evolution (FE) T1	Unfavorable evolution (UE) T1
FE T2	49 (100%)	0
UE T2	0	6 (26.08%)

**Table 7:** Variables kept into the extent multivariate logistic regression model.

Complaints	Irritability, easily angered	
	Depressed, cries easily	
	Sensation of frustration, impatience	
	Memory loss and difficulty remembering	
	Difficulty concentrating	
	Slowed thinking	

QoL	VAS global QoL	
	Physical condition	
	Brain function	
	Feelings/emotions	
	Daily life	
	Social and personal life	
	Current and future situation	

Neuropsychological tests	Low-level treatments	Trail Making Test A (TMT A) T
		Stroop C
		Stroop W
	High-level treatments	TMT B-A
		Stroop W/C
		PASAT “correct answers” (CA)
		PASAT “telescoping errors” (TE)
		Verbal phonemic fluency “M”

#### Proposal Extent Model of Multivariate Logistic Regression Model

**Figure 2:**
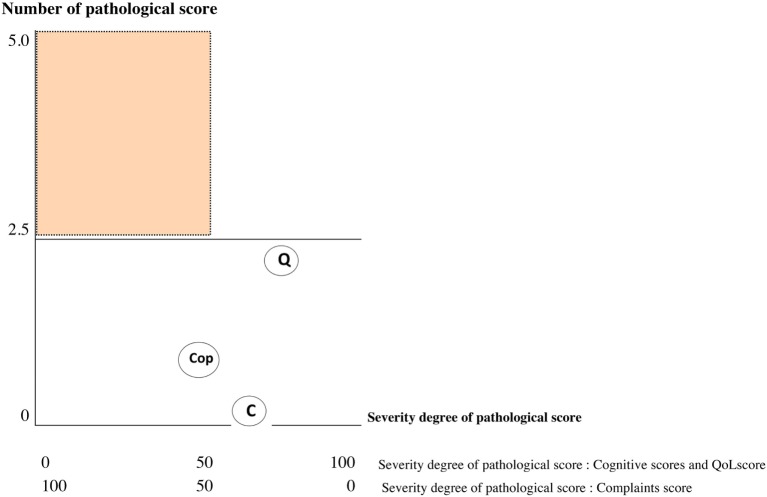
Graph showing values of each sub-score: Subject 1 [favorable evolution (FE)]. C, cognition; Q, quality of life; Cop, complaint; red score, pathological score; black score, normal score; orange area, risk zone. If one of the three coordinates is in the red area = patient identified unfavorable evolution (UE).

**Figure 3:**
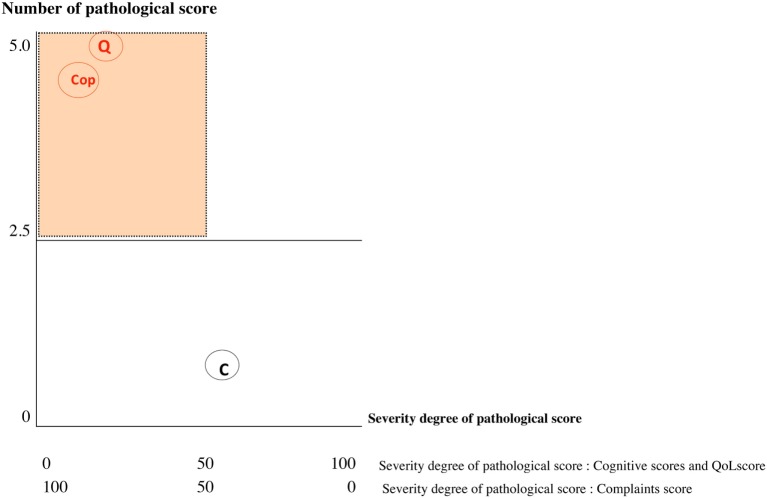
Graph showing values of each sub-score: Subject 1 [unfavorable evolution (UE)]. C, cognition; Q, quality of life; Cop, complaint; red score, pathological score; black score, normal score; orange area, risk zone. If one of the three coordinates is in the red area = patient identified UE.

Results of the multivariate logistic regression model identified three categories of variables in the domains of complaint symptoms, QoL, and cognition. To overcome the heterogeneity observed in this model (too large reliable interval size), we extend this model using variables in each category: Six complaints were retained from the RPCQ questionnaire (graded from 0 to 4), among whom mood complaint and all QoL factors of QOLIBRI were explored in addition to the VAS global QoL; for cognition, the two composite sub-scores from high and low treatment were included (Table 7). A score was obtained for each of the three categories using the calculations (Table 8 for formula; Table 9 and Figures 2 and 3 for examples).

**Table 8:** Formula and quotation of the extent multivariate logistic regression model.

Complaints	
Severity degree (/100) (cutoff ≥ 50)	– Number of complaints with a degree of disagreement >1 was identified and multiplied by 4 – Severity of complaints (0–100) was calculated using only complaints with a score >2 (0 and 1 reflecting an absence of complaint) *a* = sum of degree of disagreement >1 *b* = number of complaints with a degree of disagreement >1 [ 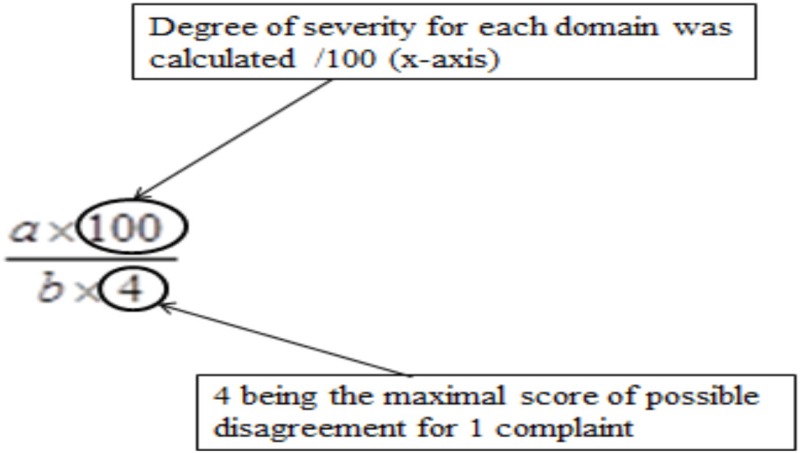 ]

Number of pathological score (/5) (cutoff ≥ 2.5)	*b* = number of complaints with a degree of disagreement >1 Maximum score on *y*-axis is /5 aximum number of complaints is /6 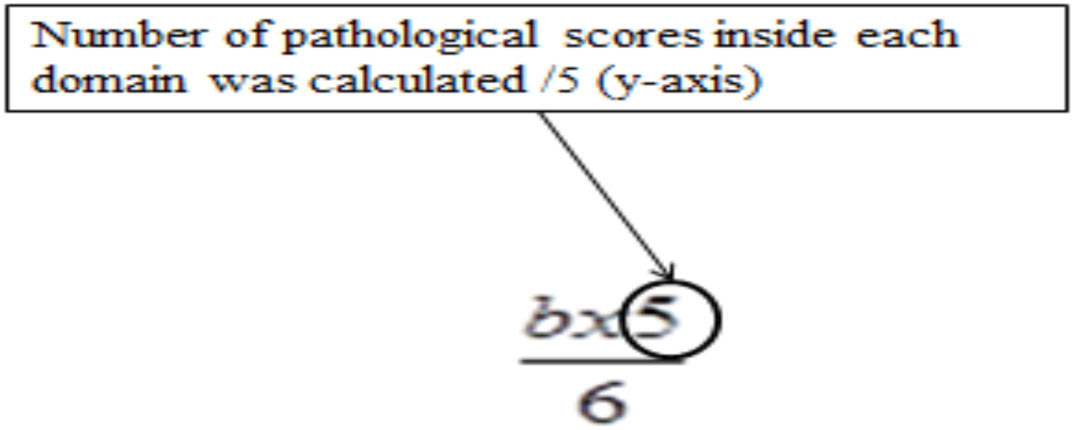

**Quality of life (QoL)**	

Severity degree (/100) (cut off ≤ 50)	The QoL score was calculated using the VAS global QoL and the Quality of Life after Brain Injury (QOLIBRI) sub-scores. Severity was calculated using the following three steps. First, we multiplied the VAS global QoL score by 10 to obtain a score out of 100, which was defined as the variable c. Next, we added the QOLIBRI sub-score s to get the Total QOLIBRI score (maximum score is 30), and applied the formula: $\frac{\left(\text{Total QOLIBRI}\times 100\right)}{30}$ to obtain a score out of 100, defined as the variable *d*. Finally, to obtain a level of severity, we calculated the mean of *c* and *d*: $\text{Severity of QoL}=\frac{c+d}{2}$

Number of pathological score (/5) (cut off ≥ 2.5)	*Number of pathological score* was calculated by considering 7 scores (1 = VAS global Qol and 6 = QOLIBRI sub-scores). QOLIBRI scores ≤3 and VAS global QoL scores ≤5 as pathologic *Number of pathological score* was calculated by adding the number of QOLIBRI scores ≤3, and then adding 1 to that sum if the VAS score was ≤5 (pathological score) 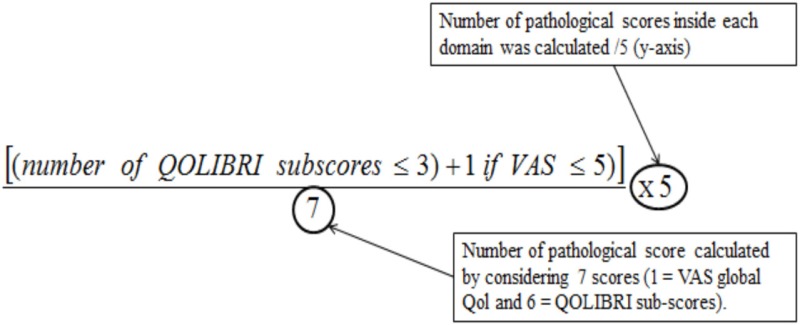

**Cognition**	
Severity degree (/100) (cutoff ≤ 40)	Regarding the cognitive domain, the score was calculated using the mean of *T*-score for each composite scores (high to low levels). *e* = mean low level (*T*-scores of the composite score “low level treatments” TMT A, Stroop Color naming, and Stroop Word reading) *f* = mean high level (TMT B-A, verbal phonemic fluency “M,” PASAT CA, PASAT TE, and Stroop Word and Color naming) $\frac{e+f}{2}$

Number of pathological score (/5) (cut off ≥ 2.5)	Number of cognitive pathological score was calculated using the established standard that a T score ≤40 is considered pathologic Maximum score on y-axis is/5 Total number of cognitive sub-score is /8 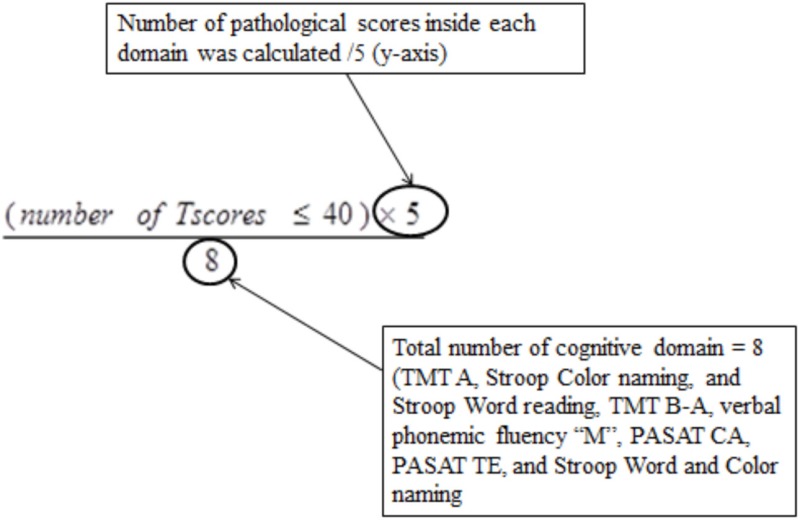

**Quotation**	
Sub-score obtained on each dimensions are represented by 2 coordinates Degree of severity for each domain (*x*-axis/100) Number of pathological scores inside each domain (*y*-axis/5) A single pathological coordinate among the three categories was adequate to classify the subject as being at risk of UE	

**Table 9:** Examples of patient classification.

Complaints	Subject 1 favorable evolution	Subject 2 unfavorable evolution
Irritability, easily angered	1	1
Depressed, cries easily	0	3
Sensation of frustration, impatience	0	2
Memory loss and difficulty remembering	2	4
Difficulty concentrating	0	4
Slowed thinking	0	4
Sum of complaints >1	2	17
Number of complaints >1	1	5
Number of complaints >1 × 4	1 × 4 = 4	5 × 4 = 20
	Number of complaints which we obtained corresponds to a score of disagreement: 2/4 (4 being the maximal score of possible disagreement for 1 complaint)	Number of complaints which we obtained corresponds to a score of disagreement: 17/20 (20 being the maximal score of possible disagreement for 5 complaints)
Severity degree: (sum of complaints (>1) × 100)/(number of complaints (>1 × 4))	(2/4) ××100 = 50	(17/20) × 100 = 85
Number of pathological score: (number of pathological score × 5)/6	(1 × 5)/6 = 0.8	(5 × 5)/6 = 4.17

**Quality of life (QoL)**		

VAS global QoL	8	2
Physical condition	2	1
Brain function	4	1
Feelings/emotions	3	1
Daily life	3	1
Social and personal life	5	1
Current and future situation	4	1
Sum QOLIBRI	21	6
Sum QOLIBRI sub-scores: (QOLIBRI total) × 100/30	70	20
Total QoL/100: VAS global QoL × 10	80	20
Severity degree: mean VAS global QoL (*c*) and QOLIBRI sub-scores (*d*): (*c* + *d*)/2	(80 + 70)/2 = 75	(20 + 20)/2 = 20
Sum of QOLIBRI pathological sub-scores ≤3 and VAS global QoL ≤5	QOLIBRI pathological sub-scores = 3	QOLIBRI pathological sub-scores = 6
	VAS global QoL pathological score = 0	VAS global QoL pathological score = 1
	Total = 3	Total = 7

Number of pathological score: [(sum of QOLIBRI pathological sub-scores ≤3 and VAS global QoL ≤5) × 5]/7	(3 × 5)/7 = 2.14	(7 × 5)/7 = 5

The 72 MTBI patients were categorized according to this approach using data from the early evaluation time point. The resulting classification was then compared with the DSM-IV classification attributed at the 6-month time point concerning the presence or absence of PCS in order to determine the reliability of our model. Statistical analyses revealed that the extent model exhibited 95.7% sensitivity and 77.6% specificity.

## Discussion

This study was designed to identify early risk factors of UE following MTBI. We compared patients with FE and UE at early and late time points, and then developed univariate and multivariate logistic regression models. All MTBI subjects presented with disturbances in all domains evaluated at the early time point. In agreement with other studies, several areas were affected at this early time point after a MTBI; the affected areas were greater in number and effect and covered all domains evaluated in the unfavorable group compared with the FE group (13, 31, 32).

The univariate logistic regression model confirmed the absence of specificity of these disturbances; the large majority of scores obtained for each variable correlated with the prognosis. These results are in agreement with studies evaluating a large range of symptoms covering different domains (5, 7, 13, 32–35).

The multivariate model was then used with the 20 variables that exhibited the most robust correlation, as identified using the univariate model (*p* < 0.01). Three groups of early prognostic factors for PCS were identified: complaints, evaluation of QoL, and cognitive function determined by neuropsychological tests. Despite strong statistical power (Table 6), the reliable intervals are too large, limiting the reliability of this classification. To overcome this finding limitation, we developed a proposal extent model using a wider range of variables, to identify MTBI at risk of UE. This extent model was able to identify 95.7% of MTBI subjects with PCS at 6 months, as defined by the DSM-IV classification.

Above all, this result confirms the importance of the subjective elements identified in the RPCQ symptom questionnaire and the QoL evaluations. Although this domain is often undervalued or ignored, we demonstrate here that it allows direct access to the level of suffering experienced by MTBI patients. Our results demonstrate that the number of symptoms and the degree of discomfort are significantly higher for the unfavorable group at the early time point, and that QoL is the more significantly altered component.

The notion of QoL covers several domains reflecting subjective perceptions by an individual. Evaluation of QoL is an indicator of interest reflecting the patient’s self-perception, which is often the only element that allows the clinician to determine differences in pre- and post-traumatic states. Emanuelson and associates (31) demonstrated this by evaluating QoL and PCS progression; they concluded that the QoL measure allows evaluation of MTBI, as it correlates strongly with the presence of PCS.

The unfavorable group exhibited greater cognitive dysfunction regarding both low-level and high-level treatments. Our multivariate logistic regression model and the extent model support the role of cognitive performance in predicting PCS. This is of particular interest, as few studies to date have confirmed the extent of early cognitive deficits in MTBI patients at risk of UE. Ponsford and co-workers (17) demonstrated that at one week and three months after MTBI, cognitive factors were not predictive of PCS, and this finding was confirmed in a meta-analysis of the literature conducted by Cassidy and colleagues (13). Nonetheless, other groups have reported cognitive difficulties to be an early marker for more difficult recovery: in both the Wojcik model (36) and the earlier model of the WHO Collaborating Centre Task Force on Mild Traumatic Brain Injury (8). These studies demonstrated that the presence of memory disturbances is a risk factor for slow recovery.

Functional MRI studies have revealed greater activation in the brain areas implicated in memory when symptoms are more severe at an earlier stage after MTBI (37, 38). Mathias and Coats (39) showed that MTBI patients performed worse than healthy volunteers in an evaluation of verbal phonemic fluency, a variable that was also identified in our multivariate logistic analysis. These results were confirmed by Goldstein and Levin in 2001 for the same test (40). The absence of specificity of cognitive deficits may be explained by the concomitant presence of alterations in attention treatments and a slowing of information processing, which may, in turn, cause a more global type of cognitive dysfunction observed from an early stage as reported by Azouvi and associates (41).

Thus, PCS appears to be dependent on a multidimensional system whose configuration can be specific from one individual to the next. This is not particularly surprising when one considers interactions as inter-dimensional, because affecting any one dimension will result in repercussions in all of the other dimensions. The Steptoe model (42) takes into account the different interactions of bio-psycho-social factors after a stressful situation, such as an accident.

It is also recognized that anxiety disorders can affect cognitive performance, such as attention processing or the coding of information by memory (43–45). A depressive state can also affect attention processing (46), slow information processing, disturb encoding functioning and the recovery of verbal information (47), and affect verbal phonemic fluency (48).

Pain, which also affects other domains, is common after MTBI (4, 33, 49). Our multivariate logistic regression model did not identify pain as a prognostic factor. This is in agreement with other studies that do not consider pain a prognostic factor of UE (11, 13, 36). Meares and co-workers (50) suggest that pain is more likely to be associated with early PCS than with persistent PCS. Therefore, our prognostic model that does not integrate pain as a factor is consistent with integrated predictive models such as the “diasthesis-stress paradigm” proposed by Wood (9), and with other earlier models such as that of the European Federation of Neurological Societies (EFNS, Task Force, 2002) (51), which reports three domains of related risk factors: the patient, the violence of the trauma, and the initial severity (51).

Our results show well the evolution of the symptoms that in the acute stage are most frequently organic disorder to evolve toward in the latest stage most frequently mood, QoL, and the cognitive functioning disorder. We also notice that the “subjective” indicators are particularly interesting to identify prematurely a risk of UE following the example of organic indicators. These results are in the same line of recent systematic review of the literature of Cassidy and associates (13). They suggest that the persistency of the symptoms would more be connected to the psychosocial factors, a number of symptoms for the acute stage, and the presence of emotional stress.

### Study Limitations

Though our patient sample was large, the number of patients categorized as having a UE was rather small, which might explain the degree of difference in our multivariate logistic regression model. So, our study could be considered as pilot and results have to be viewed with caution and need further verification and confirmation. The set of variables extracted from the two logistic regression analyses associated statistical methods with clinical experience in MTBI (i.e., we chose among statistical significant variables of every extracted domain from those models). The development of this extent model, which was prepared as a prognostic grid on the basis of a statistical model, could be considered a limitation *per se*. Nonetheless, we have identified reliable and sensitive group variables. Indeed, the specificity of this extent model was calculated to be 77.6%, indicating that some FE patients are at risk of being incorrectly diagnosed as being at risk of unfavorable progression. However, the non-diagnosis of patients potentially at risk of poor outcomes, which is critical given the serious personal and social consequences of PCS, is very limited (95.7% sensitivity).

This study was based on a cluster of tests, which required over an hour to administer. This renders it impractical for use with patients admitted for a MTBI in an emergency room setting. However, it has permitted taking a step toward an early forecast for the risk of a UE following a MTBI. Certainly, the evaluations of the complaints and the QoL, obtained *via* two self-evaluations, can be performed by the patient in the waiting room, for example, and does not take time away from healthcare personnel. Only the neuropsychological evaluation need be performed by a healthcare professional, and it is relatively short, taking at most 20 min. The next step will be to design a short checklist of symptoms and tests which could be filled out in a few minutes to select those at risk of PCS and direct them to a specific treatment and to a second examination in the ensuing weeks in an attempt to prevent persistent PCS.

## Conclusion

Post-concussive syndrome is a complex condition. Our results demonstrate the need for a multidimensional approach based on cognitive, somatic/mood, and QoL features in order to ensure the initial care of MTBI patients and the early identification of PCS. In this study, prognostic categories included the patients’ symptoms, QoL, and cognitive performances. The primary objective of this study, to identify early predictive factors of higher risk of UE in order to establish an effective diagnostic tool, was attained via the design of a prognostic grid. The prognostic tool devised will allow systematic evaluation of MTBI patients with respect to several features in order to rapidly identify the risk of developing PCS, both qualitatively and quantitatively, and customize care accordingly. It will also assist with further research into the efficacy of early care for MTBI patients.

## Ethics Statement

The study was approved by the Ethics Committee of the Pitié-Salpêtrière University Hospital (ID RCB: 2008-A01542-53, Paris, France).

## Author Contributions

All authors were involved in the drafting of the manuscript or revising it critically for important intellectual content to include the final approval of this version for publication. SC, SB, NA, SM, and MM substantially contributed to the conception and design of the work, and analysis and interpretation of data. SC, SB, and NA substantially contributed to the acquisition of data. All agreed to be accountable for all aspects of the work in ensuring that questions related to the accuracy or integrity of any part of the work are appropriately investigated and resolved.

## Conflict of Interest Statement

The authors declare that the research was conducted in the absence of any commercial or financial relationships that could be construed as a potential conflict of interest. There were no competing financial interests. Funding from GMF excluded any recommendations or restraints regarding methodology, results, and publications.
